# Abortion in Japan

**Published:** 1876-02

**Authors:** 


Abortion in Japan.—According to Hofman (Contributions of the German Society for Sociology and Ethnology, Yokohama, 1875) induced abortion is not permitted by law in Japan, and those guilty of producing the same are disgraced. It is met with in those unlawfully pregnant and among the lower classes, and is induced by women especially fitted for the purpose. A piece of the root of the *achyrandes aspera* a foot in length is introduced into the uterus between its walls and the envelope of the fetus, and is allowed to remain from one to two days. The root is first covered with musk, which is also administered internally. Success always attends this procedure. An introduction of a piece of silk impregnated with musk is also occasionally resorted to. Sometimes even the forcible introduction of a sharp piece of bamboo into the cavity of the uterus is heard of, but is generally fatal in its results. The most favorable time is during the fourth and fifth months of pregnancy.

